# The efficacies and biomarker investigations of anti-programmed death-1 (anti-PD-1)-based therapies for metastatic bone and soft tissue sarcoma

**DOI:** 10.20892/j.issn.2095-3941.2021.0270

**Published:** 2021-11-25

**Authors:** Jia Lu, Ting Li, Zhichao Liao, Hui Yu, Yongtian Zhao, Haixiao Wu, Zhiwu Ren, Jun Zhao, Ruwei Xing, Sheng Teng, Yun Yang, Xiangchun Li, Kexin Chen, Jonathan Trent, Jilong Yang

**Affiliations:** 1 Department of Bone and Soft Tissue Tumor, Tianjin Medical University Cancer Institute & Hospital, National Clinical Research Center for Cancer, Key Laboratory of Cancer Prevention and Therapy, Tianjin, Tianjin’s Clinical Research Center for Cancer, Tianjin 300060, China; 2 Department of Infection Management, Tianjin Medical University Cancer Institute & Hospital, National Clinical Research Center for Cancer, Key Laboratory of Cancer Prevention and Therapy, Tianjin, Tianjin’s Clinical Research Center for Cancer, Tianjin 300060, China; 3 Key Laboratory of Molecular Cancer Epidemiology, Tianjin Medical University Cancer Institute & Hospital, National Clinical Research Center for Cancer, Key Laboratory of Cancer Prevention and Therapy, Tianjin, Tianjin’s Clinical Research Center for Cancer, Tianjin 300060, China; 4 YuceBio Technology Co., Ltd., Shenzhen 518172, China; 5 Department of Epidemiology and Biostatistics, Tianjin Medical University Cancer Institute & Hospital, National Clinical Research Center for Cancer, Key Laboratory of Cancer Prevention and Therapy, Tianjin, Tianjin’s Clinical Research Center for Cancer, Tianjin 300060, China; 6 Sarcoma Multidisciplinary Program, Sylvester Comprehensive Cancer Center, The University of Miami, Miami, FL 33136, USA

**Keywords:** Sarcoma, PD-1, PD-L1, immunotherapy, angiogenesis, safety, efficacy, biomarker

## Abstract

**Objective:**

Sarcomas are a group of rare malignancies with various subtypes. Patients with metastatic sarcoma who have failed traditional treatments can possibly achieve better prognoses from using novel therapies, including anti-programmed death-1 (PD-1)-based therapies.

**Methods:**

We retrospectively analyzed clinical data of 24 metastatic sarcoma patients from June 15, 2016 to December 30, 2019. These patients mainly received angiogenesis inhibitors combined with anti-PD-1 therapy after they became resistant to traditional treatments. Furthermore, 8 patients underwent panel DNA and whole transcript sequencing.

**Results:**

Six patients received 2 cycles of anti-PD-1 therapy and were included in the safety evaluation only group. The median follow-up time was 5.77 months. The median progression-free survival was 7.59 months, the overall response rate was 16.7% and the disease control rate was 55.6%. Based on whole exome and transcript sequencing data, there was no association between TMB, TNB, MSI, HLA-LOH, and PD-L1 expressions and sarcoma types with clinical responses. Immunotherapy efficacy and bioinformatics analyses indicated higher intratumoral heterogeneity (ITH) in progressive disease (PD) patients and lower ITH in partial response (PR) and stable disease patients. A higher percentage of immune cell infiltration, especially monocytes, was observed in PR patients. Active stromal gene expression was increased in PD patients but decreased in PR patients. Enrichment analysis revealed that an increased TGF-β signaling pathway was reversely correlated with anti-PD-1 efficacy, while a decreased inflammatory response signaling pathway was positively correlated with anti-PD-1 efficacy.

**Conclusions:**

Our study showed PD-1 inhibitors combined with anti-angiogenesis agents were effective and well-tolerated. ITH, monocyte ratio, stroma subtypes, and the status of immune-associated signaling pathways may be related with anti-PD-1 based therapy.

## Introduction

Sarcomas are rare tumors originating from mesenchymal tissue and account for approximately 1% of all adult malignancies, and 15% of all pediatric malignancies^1,2^. Based on their histological characteristics, the World Health Organization (WHO) classifies sarcomas into more than 100 subtypes, of which soft tissue sarcoma is divided into more than 50 subtypes^3^. Over 16,730 patients were diagnosed with bone and soft tissue sarcomas in USA in 2020, and approximately 7,070 patients have died of this disease^2,4^. The National Central Cancer Registry of China has estimated that there were 28,000 newly diagnosed bone sarcoma in China in 2015 and 20,700 deaths from this disease^5^. Approximately 39,900 new soft tissue sarcoma cases occurred nationwide in China in 2014, accounting for 1.05% of the overall cancer incidence. However, the mortality data still remains unknown. Although surgery, chemotherapy, radiotherapy, targeted therapy, and neoadjuvant therapy comprise the traditional treatments for sarcomas, they do not significantly improve the overall survival (OS), especially in patients with advanced stages. Thus, patients with stage IV sarcomas usually have a poor prognosis and cannot benefit from systemic therapy, with a median OS (mOS) time of approximately 12 months, and a 5-year survival of <10%^6–8^.

Recently, the use of immune-suppressive checkpoint inhibitors has facilitated better outcomes for patients with advanced sarcomas. Programmed death-1 (PD-1), a transmembrane protein on the surface of T cells, is an important inhibitory receptor and an important immunosuppressive molecule for the maintenance of autoimmune tolerance. As the major ligand of PD-1, programmed death-ligand 1 (PD-L1) is overexpressed in malignant tumor cells and expressed in antigen-presenting cells, lymphocytes, hematopoietic cells, and epithelial cells^9,10^. The interaction of PD-1 with PD-L1 suppresses T cells and blocks T cell attack on tumors. Tumor immunotherapy strategies that block PD-1 and/or PD-L1 have been shown to be effective in various malignancies, such as melanomas, lung cancers, and lymphoma^11–13^.

Although anti-PD-1 therapy is effective in other tumor types, clinical trials of anti-PD-1 therapies for sarcomas are still ongoing and are not currently being used in clinics. Groisberg et al.^14^ analyzed the medical records of patients with advanced sarcomas referred to MD Anderson Phase I clinic, who received immunotherapeutic [checkpoint inhibitors (anti-PD1, anti-PD-L1, anti CTLA4, etc.) vaccines and cytokine-based therapies]. The results showed a median overall survival (OS) of 13.4 months (a target of 11.2 months was not achieved) and a median progression-free survival (mPFS) of 2.4 months [95% confidence interval (CI: 1.9–3.2 months)]. The best response in 2 patients with alveolar soft partial sarcoma (ASPS) was a partial response (PR)^14^. In SARC028 (NCT02301039), 40 patients (*n* = 84) with soft tissue sarcoma could be evaluated for responses; the overall response rate (ORR) was 18%, the mPFS was 18 weeks (95% CI: 8–68 weeks), and the mOS was 49 weeks (95% CI: 34–73 weeks). Of these cases, patients with undifferentiated pleomorphic sarcomas (UPSs) [4 of 10 (40%)] and dedifferentiated liposarcoma (LPS) [2 of 10 (20%)] had higher responses to anti-PD-1 therapy^15^. To further confirm the clinical efficacies of pembrolizumab in UPS and LPS patients, the study created 2 expansion cohorts with advanced UPSs and LPSs. The UPS cohort achieved its primary endpoint (ORR: 23%; mPFS: 3 months; and mOS: 12 months)^16^. The efficacy of pembrolizumab in UPS patients therefore deserves further evaluation in a randomized study. However, the efficacy of pembrolizumab was not confirmed in the LPS cohort (ORR: 10%; mPFS: 2 months; and mOS: 13 months)^16^. The Alliance A091401 study was a multicenter, open, non-comparative, randomized phase II clinical study of nivolumab combined with ipilimumab for unselected patients with advanced sarcoma after multiline treatment. The patients in the trial achieved an objective remission of 16%, which was comparable to the efficacy of standard chemotherapy^17^. In the same clinical trial, the objective remission with nivolumab was 5%^17^.

The overall efficacy of anti-PD-1 therapy alone is approximately 20% for most solid tumors. Many studies have been conducted using clinical trials to identify more effective combination therapies based on PD-1 inhibitors, such as combining anti-PD-1 therapies with chemotherapy, radiotherapy, or targeted therapies. Given the role of VEGF in cancer treatment, Wilky et al.^18^ evaluated the efficiency of the VEGF receptor tyrosine kinase inhibitor, axitinib, plus the PD-1 inhibitor, pembrolizumab, in patients with sarcomas. The results showed that the ORR was 21.9%, the mPFS was 4.7 months, and the 3-month PFS was 65.6% (95% CI: 46.6–79.3) (*n* = 33). In addition, the majority of responses occurred in patients with ASPSs. The percentage of patients who achieved a clinical benefit was 72.7% (*n* = 8; 95% CI: 32.3–92.7)^18^. In a single-arm, open-label phase 2 clinical trial of apatinib in combination with carrilizumab (anti-PD1 therapy, SHR-1210) for osteosarcomas that progressed after advanced chemotherapy, the results showed a 6-month PFS of 50.9% (95% CI: 34.6%, 65.0%) at a median follow-up of 48.3 weeks. The final objective response rate was 20.9% (9/43)^19^. Patients with PD-L1 expressions ≥5% and with lung metastases tended to have a longer PFS than other patients (*P* = 0.004 and 0.017, respectively).

As studies on immunotherapy have become more extensive and complicated, corresponding clinical trials of immunotherapy-based combination therapies are attracting more attention from researchers. However, due to the low incidences of bone and soft tissue sarcomas in China, as well as the complex subtype classification, clinical research results on the immunotherapies for these disorders have been insufficient. Moreover, although PD-L1 expression, microsatellite instability (MSI), and tumor mutational burden (TMB)/tumor neoantigen burden (TNB) are recognized predictors of solid tumors, they vary greatly in sarcomas, even among different subtypes. Biomarkers for sarcoma immunotherapies have therefore still not been identified.

In this study, we described a clinical study that retrospectively analyzed several patients with distant metastases from bone and soft tissue sarcomas treated with anti-PD-1-based therapy after evaluations of efficacy and safety using conventional therapies. The results of the study showed an ORR of 22.2% (4/18), a disease control rate (DCR) of 72.2% (13/18) , and a progression-free rate (PFR) of 49.8% at 12 weeks. The mPFS was 7.59 months. Our study further reported that PD-1 inhibitor-based therapy was effective in patients with advanced sarcomas. More importantly, we found that intratumoral heterogeneity (ITH), monocyte ratio, stromal subtype, and the status of immune-related signaling pathways may be associated with the efficacy of anti-PD-1-based therapies in sarcoma patients. These results supported our establishment of the NCT04126993 clinical trial in advanced bone and soft tissue sarcomas, which will hopefully provide evidence of using anti-PD-1 based comprehensive therapy for patients with advanced sarcomas.

## Materials and methods

### Patient information and treatment

We retrospectively summarized the demographic and clinical information of 24 metastatic bone and soft tissue sarcoma patients who received anti-PD-1 monotherapy or an angiogenesis inhibitor combined anti-PD-1 therapy at the Tianjin Medical University Cancer Institute & Hospital. The patients were diagnosed from June 2016 to December 2019. Before anti-PD-1 treatment, the 24 patients had received up to 3 previous lines of systemic anticancer therapy, such as radiotherapy and chemotherapy. Chemotherapy mainly included the AI regimen, MAID regimen, and variations based on the ADM regimen. The inclusion and exclusion criteria of patients are in **Supplementary materials**. This retrospective investigation was conducted in accordance with the Declaration of Helsinki and was approved by the Ethics Committee of Tianjin Medical University Cancer Institute & Hospital (Approval No. E2019144). All followed patients provided signed informed consents. The trial registration was NCT04126993.

Detailed information of anti-PD-1 based therapies are shown in **Table 1**. The anti-PD1 inhibitor, pembrolizumab, was given by intravenous infusion over 30 min at a dose of 3 mg/kg every 3 weeks. Another anti-PD-1 inhibitor, camrelizumab, was also given by intravenous infusion at the dose of 3 mg/kg every 3 weeks. A total of 20 patients were given anti-PD-1 therapy combined with apatinib, an angiogenesis inhibitor. Apatinib was administered at a starting dose of 500 mg daily. If intolerance appeared in patients, the dose was reduced to a maximum of 50%, to 375 mg and then to 250 mg, if necessary. Patients who could not tolerate the dose of 250 mg stopped receiving treatment.

**Table 1:** The baseline characteristics of patients

Characteristics	*n* (%)
Age	
Average	43 years
Range	15–78 years
Gender	
Male	14 (58)
Female	10 (42)
ECOG performance status	
0	3 (12.5)
1	15 (62.5)
2	5 (20.8)
3	1 (4.2)
Tumor type	
Soft tissue sarcoma	17 (70.8)
Synovial sarcoma	3
Liposarcoma	4
UPS	3
Rhabdomyosarcoma	2
Leiomyosarcoma	2
Clear cell sarcoma	2
MPNST	1
Non-soft tissue sarcoma	7 (29.2)
Osteosarcoma	5
Chondrosarcoma	2
Primary location	
Extremity	12 (50.0)
Abdominal/pelvis	2 (8.3)
Chest	2 (8.3)
Axial	8 (33.4)
Treatment strategy	
Mono pembrolizumab	3
Camrilizumab combined with apatinib	20
Pembrolizumab combined with radiotherapy	1

ECOG, Eastern Cooperative Oncology Group; UPS, undifferentiated pleomorphic sarcoma; MPNST, malignant peripheral nerve sheath tumor.

### Efficacy and safety evaluations

We collected information from 24 patients receiving pretreatments, including the results of physical examinations, clinical blood counts, blood chemistry panels and computed tomography scans of the measurable lesions at baseline. Toxicity was assessed monthly by medical records or by telephone call follow-ups. Measurable lesions were first assessed by computed tomography at 8 weeks, then confirmed at 12 weeks, and further evaluated every 2 months. The patients were followed-up until death, loss to follow-up, or the end of the observation period.

Clinical responses to the treatments were evaluated according to Response Evaluation Criteria in Solid Tumors 1.1 (RECIST 1.1)^20^. Evidence of efficacy was agreed upon by two independent radiologists, who were blinded to the treatments. Each patient had at least 1 measurable extracranial lesion, and the responses were evaluated according to RECIST 1.1^21,22^. Some sarcoma patients with non-measurable lesions were also evaluated according to RECIST 1.1^21,22^. Non-measurable lesions included small lesions (longest diameter <10 mm or pathological lymph nodes with 10–15 mm short diameters) as well as truly non-measurable lesions. Lesions considered truly non-measurable included leptomeningeal disease, ascites, pleural or pericardial effusion, lymphangitis involvement of the skin or lung, or abdominal mass/abdominal organomegaly that were identified by physical examination but were not measurable by reproducible imaging techniques^20–22^. Patients with these non-measurable lesions were evaluated as having a complete response (CR), progressive disease (PD), or non-CR/non-PD according to RECIST 1.1^21,22^. Non-CR/non-PD was preferred over SD (stable disease) for the patients with no target diseases^21,22^. To simplify and unify the evaluations, we replaced non-CR/non-PD with SD in some patients with non-measurable lesions.

To accurately assess the biological activities and side effects of treatments, we defined PFS as the primary endpoint, and PFR, ORR, and DCR at 12 weeks as the secondary endpoints. PFS was defined as the time from initiating anti-PD-1 treatment until disease progression according to RECIST 1.1. Disease control was defined as CR, PR, or SD. The ORR was calculated as follows: (CR+PR)/the total number of cases × 100%. The DCR was calculated as (CR+PR+SD)/the total number of cases × 100%.

All the patients were included in the safety and toxicity analyses, using medical records or telephone interviews. Treatment-related adverse events (AEs) were assessed and graded based on the (National Cancer Institute Common Terminology Criteria for Adverse Events, version 3.0)^23^.

### Immunohistochemistry (IHC) of PD-L1 expression

**Figure 1:**
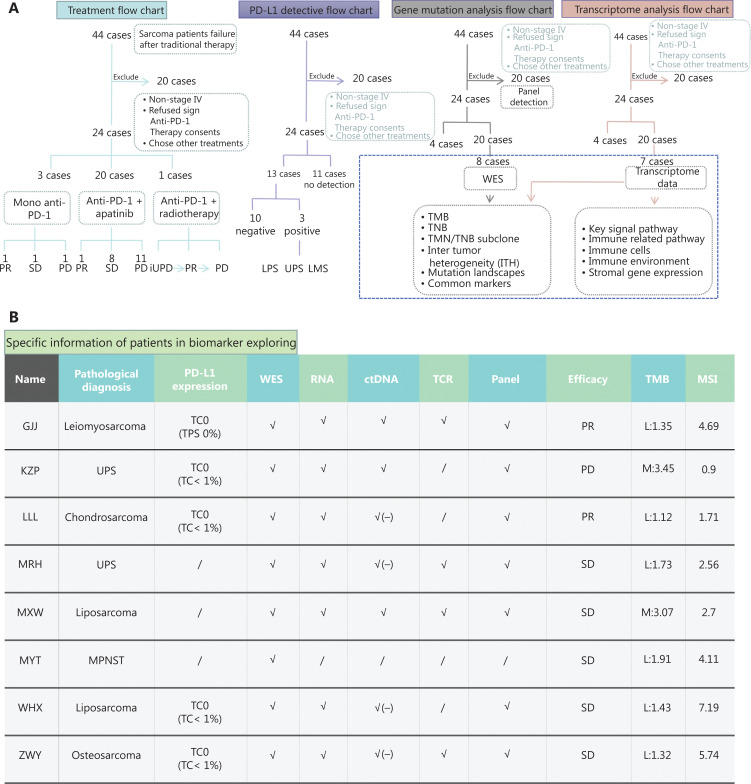
The flow chart of patient selection, PD-L1 expression, and bioinformatics analysis. (A) The flow charts and treatment flow chart. We selected 24 cases in this retrospective analysis. The treatment strategy included mono anti-PD-1 therapy (*n* = 3), anti-PD-1 + apatinib combined therapy (*n* = 20), and anti-PD-1 + radiotherapy (*n* = 1). The PD-L1 detective flow chart: among 24 patients, only 13 patients received PD-L1 expression testing. The positive ratio was 23% (3/13). The gene mutation flow chart: the large mutation landscape was composed of the panel detection data of 20 cases and whole exome sequencing (WES) data of 8 cases. We also used bioinformatics analyses, such as some biomarkers, using WES data. The transcriptome analysis flow chart: we had accompanied studies using transcriptome data of 7 cases, including key signal pathways and the immune microenvironment. (B) Specific information of patients in biomarker exploring. Before treatment, there were 8 patients with whole exon sequencing, 7 patients with RNA sequencing, and 8 patients with panel sequencing data.

PD-L1 expression was assessed in selected patients who had available tissues for testing. Some patients (*n* = 13) received biomarker testing by the Yuce Bio Company (Guangdong, China) (**Figure 1A**). This company performed the VENATA PD-L1 SP263 Assay using the Ventana BenchMark Ultra OptiView DAB IHC detection kit (Thermo Fisher Scientific, Waltham, MA, USA) with pathological tissue sections analyzed from formalin-fixed, paraffin-embedded tissue sections. The evaluation results used the index of %TC (the percentage of PD-L1 expressing tumor cells of any intensity) and %IC (the percentage of tumor area occupied by PD-L1 expression tumor-infiltrating immune cells of any intensity).

### Bioinformatics analysis of DNA and RNA sequencing data

The data of gene sequencing were obtained with tissue samples from 8 patients. Samples from all 8 patients were analyzed by whole exome sequencing (WES) and transcriptome data were obtained from 7 patients (**Figure 1A, B**). The procedures of gene sequencing were conducted by the Yuce Bio Company. Genomic DNAs from formalin fixed, paraffin embedded (FFPE) sections, from biopsy samples, or from whole blood control samples, were extracted using the Gene Read DNA FFPE Kit (Qiagen, Germantown, MD, USA) and the Mag-Bind Blood & Tissue DNA HDQ 96 Kit (Qiagen), respectively. Library preparations were performed with KAPA Library Quantification Kit (Roche, Indianapolis, IN, USA), the target enrichment was performed using the Target Seq Enrichment Kit (iGene Tech, Beijing, China) and the sequencing was performed on a NovaSeq (Ilumina, San Diego, CA, USA).

The raw reads of WES-seq were processed by SOAPnuke (version 1.5.6, parameters: -l 20 -q 0.1 -n 0.1) to remove ambiguous reads and/or low quality reads. These qualified sequence reads were then aligned to the human reference genome (UCSC hg38) using BWA-mem (BWA, version 0.7.12). Removal of duplicates by SAMBLASTER (Version 0.1.22) was used to reduce biases in downstream analyses. The single nucleotide variants were detected using VarScan, version 2.4. Single-nucleotide variants (SNVs) with an allele read count of less than 20 or with corresponding normal coverage of less than 20 reads were removed. The mutations were then filtered using a customized Perl script to eliminate false positives and annotated by SnpEff, version 4.3. TMB was calculated using non-silent somatic mutations, including coding base substitutions and indels. To analyze human lymphocyte antigen-I typing across patients, we followed the method developed by Yi^24^. All non-silent mutations were translated to 9–11 mer peptides, which were used to predict potential neoantigens, as previously described^25^. Tumor neoantigen burden (TNB) was measured as the number of mutations that were predicted to generate neoantigens per megabase.

The cancer cell fraction (CCF) of mutations was estimated using PyClone, version 0.13.0 with the tumor purity estimated by All-FIT. The ratios of subclone mutations to all mutations were interpreted as ITH. Microsatellite instable (MSI) scores were analyzed by interrogating 344 available genomic microsatellites using MSIsensor, Version 0.2. Tumor samples with MSI scores >20 were defined as MSI-H.

For raw reads of RNA-seq, preprocessing was conducted as described above. Clean data were then aligned on the hg19 genome using the STAR aligner with default parameters. Aligned reads were then counted using RESM. All differential gene analysis was conducted using the DESeq2 package. Differentially expressed genes (DEGs) were considered for further analysis with a *P*-value <0.05. The estimation of immune populations was done using the QuanTIseq algorithm.

### Statistical analysis

All statistical analyses were conducted using Statistical Package for the Social Sciences (SPSS) software, Version 21.0 as previously described^26^. We observed and summarized the objective response based on the best response while on treatment. We used the Life Table method to estimate overall and progression-free survivals. Using the Kaplan-Meier method, we estimated the single factor effect. The ORR and DCR analyses were based on frequencies.

## Results

### Efficacies and safety evaluations of anti-PD-1 based therapies

#### Maximum change in target lesion size

**Figure 2:**
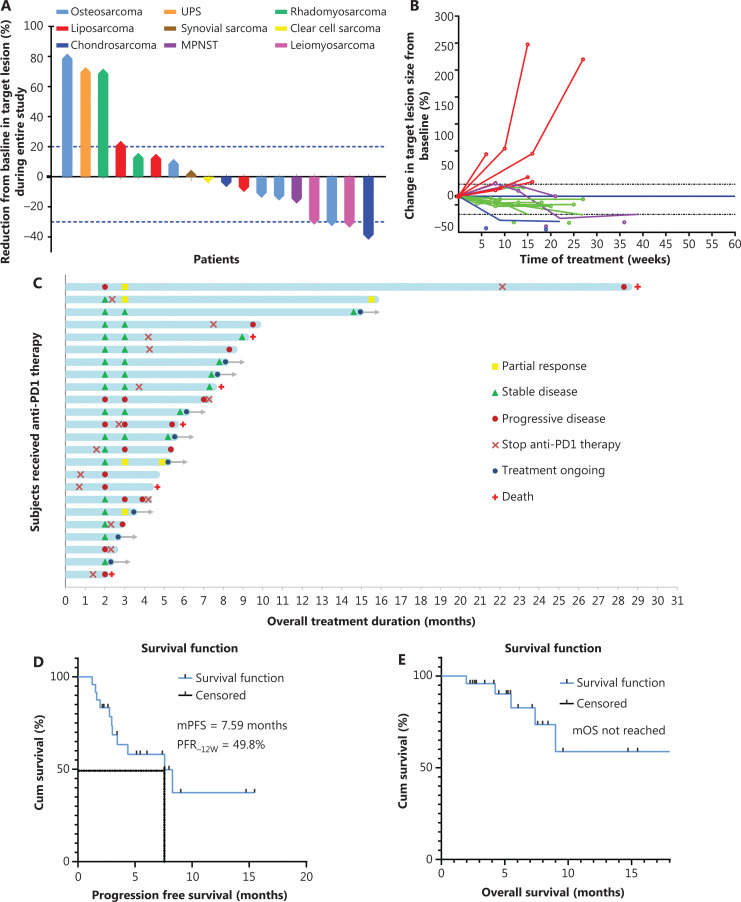
Efficacy of treatment based on PD-1 inhibitors in patients with sarcomas. (A) Maximum changes in target lesions in patients with stage IV sarcomas who were treated with PD-1 inhibitor-based therapy. A total of 14 patients (77.8%, 14/18) responded to the treatment, no patients achieved complete remission, 4 patients (22.2%, 4/18) achieved partial remission, 10 patients (55.6%, 10/18) achieved stable disease, and 4 patients (22.2%, 4/18) suffered from PD. Up to 77.8% (14/18) of patients had responses to anti-PD-1-based treatments. (B) Changes of the target lesions in 18 patients with measurable lesions. Red lines: target lesions increased ≥20% from baseline; purple lines: target lesions initially increased ≤ 20% and shrank by 0%–40%; green lines: target lesion changes shrank by 5%–30%; and blue lines: target lesions shrank ≥ 30%. (C) Overall responses of 24 patients with stage IV sarcomas. The patients received a treatment of mono PD-1 inhibitor with or without apatinib. By the end of the observation period, 6 patients died from progressive disease. (D, E) The survival curves of progression-free survival (PFS) and overall survival (OS) in patients with anti PD-1 based treatment. The median PFS was 7.59 months and the median OS was not reached.

First, we evaluated the optimal response to anti-PD-1-based therapies. The maximum change in target lesion size was evaluated according to RECIST 1.1. Of these 24 patients, 6 patients received only 2 cycles of anti-PD-1 therapy, so data from only 18 patients were included in this evaluation. As a result, no patient achieved CR, 4 patients (22.2%, 4/18) achieved PR, 10 patients (55.6%, 10/18) achieved SD, and 4 patients (22.2%, 4/18) were PD at the time of best efficacy evaluation. Thus, up to 77.8% (14/18) of the patients responded to anti-PD-1-based therapies during the treatment period (**Figure 2A**). There was no significant difference in maximum change of tumor size between bone sarcomas (osteosarcomas and chondrosarcomas) and soft tissue sarcomas (**Supplementary Figure S1A–B**).

#### Clinical responses at 12 weeks

Among all 24 patients, 6 patients received only 2 cycles of anti-PD-1 therapy, and the remaining 18 patients (18/24, 75%) were assessed with efficacy at 8 weeks and then confirmed at 12 weeks (**Table 2**). As shown in **Table 2** and **Figure 2B–C**, no patient achieved CR, 4 patients achieved PR (22.2%, 4/18), and 9 patients achieved SD (50%, 9/18) at the 12 week evaluation. The target lesions of 5 patients increased by 20% and were evaluated as PD (27.8%, 5/18), including 1 patient who died of disease progression (**Figure 2B–C**). The 12-week ORR was 22.2% (4/18), the DCR was 72.2% [(4 + 9)/18], and the PFR was 49.8% at 12 weeks (**Figure 2B–C**; **Table 2**).

**Table 2:** Clinical response to anti-PD-1-based therapy in metastatic sarcomas

Response	12W	Overall response
CR	0	0
PR	4	3
SD	9	7
PD	5	8
Excluded	6	6
ORR	22.2% (4/18)	16.7% (3/18)
DCR	72.2% (13/18)	55.6% (10/18)
	PFR_−12W_ = 49.8%	mPFS = 7.59 month
		mOS unreached

CR, complete response; PR, partial response; SD, stable disease; PD, progressive disease; DCR, disease control rate; ORR, objective response rate; PFR, progression-free survival; mPFS, median progression-free survival.

At 12 weeks, the DCR was 71.5% (5/7) and the ORR was 28.5% (2/7) for bone sarcomas. For soft tissue sarcomas, the DCR was 72.7% (8/11) and the ORR was 18.3% (2/11). There were no significant differences between bone sarcomas and soft tissue sarcomas in DCR, ORR, and PFR using the chi-square test (*P* > 0.05).

#### Overall response

After reviewing all the clinical data of the 18 sarcoma patients who had enough information for the final efficacy evaluations, 3 patients achieved PR, 7 patients achieved SD, 8 patients had PD, and 4 patients died of PD (**Table 3 and Figure 2C**). Finally, the ORR was 16.7% (3/18) and the DCR was 55.6% (10/18) (**Table 2**). The median PFS was 7.59 months, and the median OS was not reached (**Figure 2D, 2E**). However, there was no significant difference between bone sarcomas and soft tissue sarcomas (**Supplementary Figure S1C–D**). Detailed treatment information for leiomyosarcoma patients treated with anti-PD-1 in combination with radiotherapy and osteosarcoma patients treated with anti-PD-1 monotherapy are listed in **Supplementary Figures S3–4**.

**Table 3:** Response of all patients to treatment based on PD-1 inhibitors

Patient	Pathologic type	Treatment	8w	12w	Overall	PD-L1	MSI
LYC	OS	Pembrolizumab	SD	PR	PR	−	MSS
WJH	OS	Combined	SD	SD	SD	−	
XX	OS	Combined	SD	SD	SD		
ZWY	OS	Combined	PD	PD	PD	−	5.74
LY	OS	Combined	SD	PD	PD		
ZFL	LPS	Combined	SD	SD	PD	−	
WHX	LPS	Combined	SD	SD	SD	−	7.19
XMC	LPS	Combined	PD			−	
MXW	LPS	Combined	SD			+	2.7
ZX	SS	Pembrolizumab	SD	SD	SD	−	
WY	SS	Combined	SD	SD	SD		
TMM	SS	Combined	SD	SD	SD		
DLY	UPS	Combined	PD	PD	PD		
MRH	UPS	Combined	SD			+	2.56
KZP	UPS	Combined	PD			-	0.9
CMJ	RMS	Combined	PD				
GXS	RMS	Combined	SD	PD	PD		
YHB	CDS	Combined	SD	SD	SD		
LLL	CDS	Combined	SD	PR	PR		1.71
SHY	LMS	Pembrolizumab+radiotherapy	PD	PR	PD	+	
GJJ	LMS	Combined	SD	SD	SD	−	4.69
PFL	CCS	Pembrolizumab	PD				
WB	CCS	Combined	SD	PD	PD		
MYT	MPNST	Combined	SD	SD	PD	−	4.11

OS, osteosarcoma; SS, synovial sarcoma; RMS, rhabdomyosarcoma; CDS, chondrosarcoma; LMS, leiomyosarcoma; CCS, clear cell sarcoma; LPS, liposarcoma; MPNST, malignant peripheral nerve sheath tumor; UPS, undifferentiated pleomorphic sarcoma. Combined treatment (camrelizumab+apatinib).

#### Safety and adverse events

All 24 patients were evaluated for safety and adverse events. AEs caused by mono anti-PD-1 therapy were tolerated, and no patient withdrew during the observations. More severe AEs occurred in patients who received combined therapy (camrelizumab + apatinib). Most AEs occurred after 2 or 3 cycles of treatment. Common AEs included reactive capillary hyperplasia (RCCEP) (*n* = 7, 17.9%), fatigue and weakness (*n* = 5, 12.8%), fever (*n* = 3, 7.7%), anemia (*n* = 3, 7.7%), leukopenia (*n* = 3, 7.7%), digestive function (*n* = 3, 7.7%), hypohepatia (*n* = 2, 5.1%), pain (*n* = 2, 5.1%), hypertension (*n* = 2, 5.1%), HFSR (*n* = 2, 5.1%), and rash (*n* = 2, 5.1%). In addition, sporadic albuminuria, diarrhea, pain, limb edema, and hypothyroidism also occurred in some patients (**Table 4**). Grade 4 AEs included only hypertension, which could be controlled by treatment.

**Table 4:** Adverse events during treatments

Adverse event	Grade1*	Grade2	Grade3	Grade4	Total
RCCEP	7	0	0	0	7 (17.9%)
Fatigue and weakness	5	0	0	0	5 (12.8%)
Digestive dysfunction	3	0	0	0	3 (7.7%)
Fever	3	0	0	0	3 (7.7%)
Anemia	3	0	0	0	3 (7.7%)
Leukopenia	2	0	1	0	3 (7.7%)
Pain	2	0	0	0	2 (5.1%)
Hypertension	1	0	0	1	2 (5.1%)
Rash	2	0	0	0	2 (5.1%)
HFSR	1	1	0	0	2 (5.1%)
Hypohepatia	1	1	0	0	2 (5.1%)
Diarrhea	1	0	0	0	1 (2.6%)
Albuminuria	1	0	0	0	1 (2.6%)
Limb edema	1	0	0	0	1 (2.6%)
Anorexia	1	0	0	0	1 (2.6%)
Hypothyroidism	1	0	0	0	1 (2.6%)

*According to the National Cancer Institute Common Terminology Criteria for Adverse Events (CTCAE, version 3.0). RCCEP, reactive capillary hyperplasia; HFSR, hand and foot syndrome.

AEs were more complex in patients receiving combination therapy, when compared with those receiving single agent anti-PD-1 therapy. However, the grade was limited to grade 1 or 2. Two patients (OS and chondrosarcomas) discontinued apatinib therapy or had their dose reduced due to side effects, mainly consisting of uncontrolled wound healing. No more significant side effects were observed in patients treated in combination.

### Identification of biomarkers for anti-PD-1-based therapies

#### The genomic landscape of sarcoma patients

**Figure 3:**
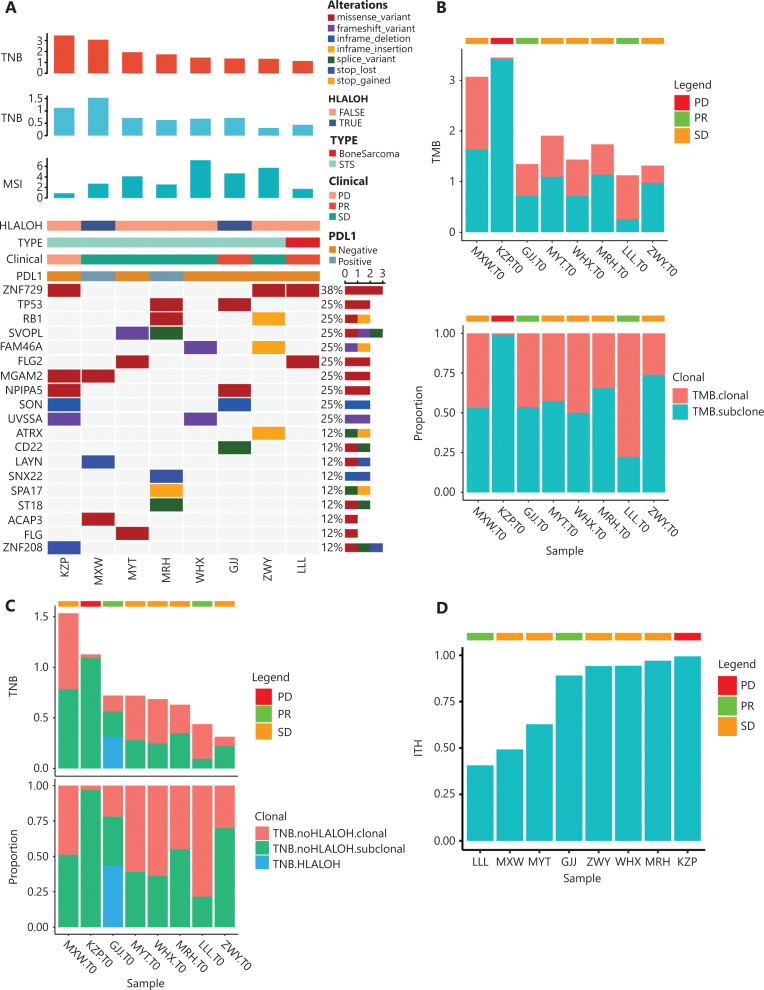
Biomarker exploring for anti-PD-1 based therapy. (A) The landscape map with whole exome sequencing data of 8 cases before treatment combined with TMB/TNB/MSI and other information. From top to bottom are as follows: TMB, TNB, MSI, HLA-LOH, or not, sarcoma subtype, efficacy, PD-L1, and the top 19 genes with the highest mutation frequency. (B) Tumor mutational burden (TMB) in 8 patients is described using a histogram. It was divided into TMB-clones and TMB-subclones. The percentage of subclones present in the percentages of neoantigens. (C) Tumor neoantigen burden in 8 patients is described using a histogram. Two patients had the phenomenon of HLA-LOH, but it had an effect on only 1 patient because half of the neoantigens could not be presented. (D) Intertumoral heterogeneity (ITH) in 8 patients using a histogram. The patients with PD showed the highest percentage of ITH.

The mutational landscape was described by pretreatment WES data from 8 patients, combining TMB, TNB, MSI, HLA-LOH, and PD-L1 expressions, sarcoma types, and anti-PD-1 clinical responses (**Figures 1B and 3A**). The top 3 genes with the highest mutation frequencies included ZNF729 (38%), TP53 (25%), and RB1 (25%), which are known drivers of soft tissue sarcomas (**Figure 3A**)^27^. As mentioned above and shown in **Figure 3**, it was difficult to find a significant association of TMB, TNB, MSI, HLA-LOH (loss of heterozygosity in human leukocyte antigens), PD-L1 expression, and sarcoma types with the clinical response of anti-PD-1-based therapy in our cohort.

By analyzing the mutated genes and clinical responses of each patient, 1 patient (KZP) carried a MDM2 amplification, which is a negative indicator associated with immunotherapy, indicating the risk of hyper progression. Overall, these results explained the patients’ non-response to anti-PD-1 therapy.

HLA-LOH refers to deletion of HLA heterozygosity. A positive HLA-LOH represents heterozygous deletion or complete loss of function of the HLA gene. Loss of function results in loss of antigen presentation and inability of the immune system to recognize the tumor. HLA-LOH is an indicator of immune escape in monotherapy, but not in immuno-chemotherapy. In our results, the HLA-LOH phenomenon occurred in patients GJJ and MXW (**Table 5**), but they both responded to anti-PD-1 treatment (PR and SD).

**Table 5:** The testing results of common biomarkers

Sample	GJJ.T0_WES	KZP.T0_WES	LLL.T0_WES	MRH.T0_WES	MXW.T0_WES	MYT.T0_WES	WHX.T0_WES	ZWY.T0_WES
DDR	–	–	–	–	–	–	–	BRIP1, p. Ile1209Met
TMB	TMB-L:1.35	TMB-M:3.45	TMB-L:1.12	TMB-L:1.73	TMB-M:3.07	TMB-L:1.91	TMB-L:1.43	TMB-L:1.32
TNB	TNB-M:0.72	TNB-M:1.13	TNB-L:0.44	TNB-M:0.63	TNB-M:1.53	TNB-M:0.72	TNB-M:0.69	TNB-L:0.31
MSI	4.69	0.9	1.71	2.56	2.7	4.11	7.19	5.74
HLA mutation	–	–	–	–	–	–	–	–
B2M mutation	–	–	–	–	–	–	–	–
ALK fusion	–	–	–	–	–	–	–	–
EGFR mutation	–	–	–	–	–	–	–	–
JAK1/2 mutation	–	–	–	–	–	–	–	–
PTEN mutation	–	–	–	–	–	–	–	–
STK11mutation	–	–	–	–	STK11, p. Met18Ile	–	–	–
MDM2 amplification	–	MDM2, amp8	–	–	–	–	–	–
MDM4 amplification	–	–	–	–	–	–	–	–
DNMT3A mutation	–	–	–	–	–	–	–	–
HLA-LOH	HLA-A*30:01							
HLA-C*03:03	–	–	–	HLA-A*24:02				
HLA-C*01:02	–	–	–					

#### PD-L1 expression, TMB/TNB, MSI, and ITH

PD-L1 expression, and TMB and MSI play important roles as predictive biomarkers for immunotherapy^28,29^. We examined PD-L1 expressions in the tissues of 13 patients with subtypes including LMS, SS, MPNST, LPS, UPS, chondrosarcoma, and OS (**Figure 1A; Table 3**). The results showed that only 3 patients (LPS, UPS, LMS; 23.07%; 3/13) had positive PD-L1 expressions without a tendency for a sarcoma type (**Figure 1B; Supplementary Figure S3J; Table 3**). Even the 3 patients with positive PD-L1 expressions achieved PR or SD efficacy, and the osteosarcoma patients with negative PD-L1 expressions achieved PR. Fisher’s exact test showed no significant correlations between PD-L1 expressions and responses to anti-PD-1 therapy in sarcoma patients (Fisher’s exact test = 4.708; *P* = 0.185; **Table 3**).

Previous studies showed that TMB values of sarcomas were usually low^30^. Consistent with these studies, the TMB was also low in all patients in our cohort, ranging from 1.12–3.45 mutations/MBs. A similar situation was detected regarding TNB (**Figures 3B–C**). Similarly, MSI was detected by next-generation sequencing (NGS) in patients with microsatellite stable (MSS) tumors. Although PD-L1 expression, TMB, and MSI are recognized predictors of prognosis and immunotherapy efficacy in solid tumors, these biomarkers did not show a better prognostic significance in our cohort. Therefore, whether PD-L1 expression, TMB, or MSI can serve as biomarkers for anti-PD-1 therapy in sarcoma remains to be determined.

ITH refers to the presence of distinct tumor cell populations, which describes the differences in morphologies and expressions of histopathological markers in different subtypes of cancer. It has been reported to be associated with poor clinical prognoses of various tumors, such as non-small cell lung cancer (NSCLC) and melanoma^31–33^. It has been reported that patients with low levels of ITH always had improved responses to immunotherapy^34^. In the present, a higher ITH was observed in PD patients. It was also observed that the PR and SD of patients had a relatively lower level of ITH based on the relationship between ITH and the efficacy of immunotherapy (**Figure 3D**).

#### Immune microenvironment

The tumor microenvironment is a complex ecosystem, composed of cancerous and non-cancerous cells, including stromal cells and immune cells^35^. The immune cells constitute the tumor immune microenvironment, and it has been reported that these tumor-associated immune cells may possess tumor-antagonizing or tumor-promoting functions^36^.

**Figure 4:**
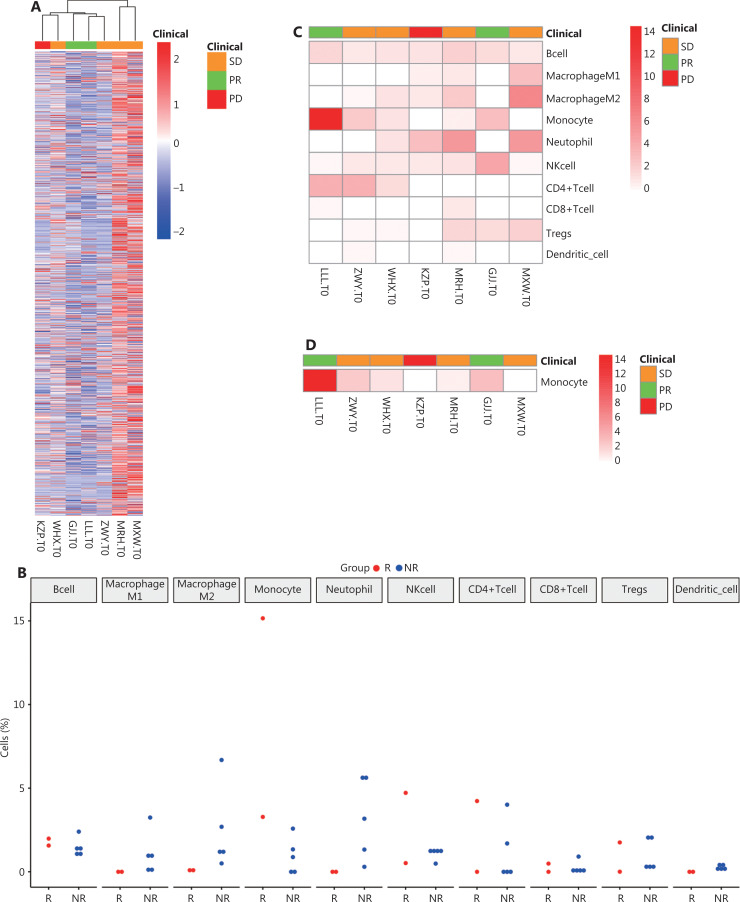

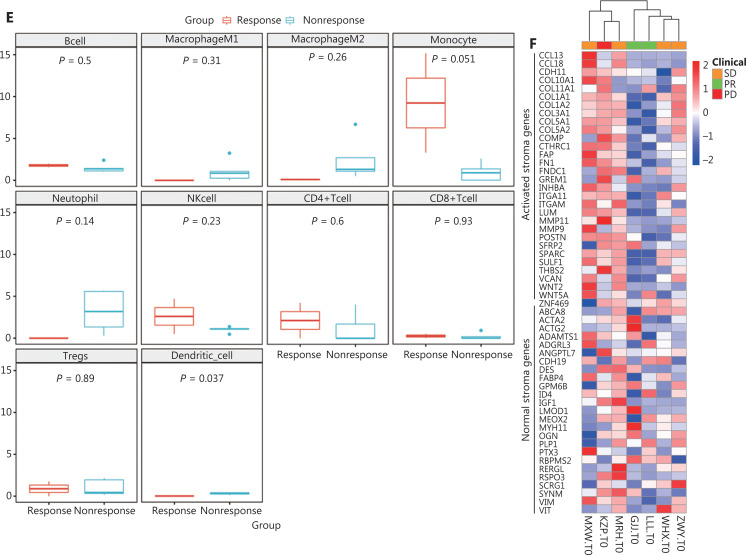
The analysis of RNA data of 7 patients. (A) Heat map of immunity-related gene expressions. (B) The analysis of infiltration percentages of tumor immunocytes. The whole cell percentages in the response (PR + SD) and nonresponse (PD) groups. (C) The heat map of immune cells in 7 patients. (D) The heat map of monocytes in 7 patients. (E) Variation analysis of the different immunocytes between the response and nonresponse groups, including B cells, M1/M2 macrophages, monocytes, neutrophils, NK cells, CD4^+^ T cells, CD8^+^ T cells, Tregs, and dendritic cells. (F) Heat map of clustering analysis of these 8 patients. The patient with apartial response showed normal stroma subtype, and patients with PD showed an activated stroma subtype according to stroma signature genes.

Using the immune-related gene set from ImmPort data, we analyzed the expressions of immune-related genes in the samples. Even though 2 PR patients had decreased expressions of immune-related genes, clustering analysis revealed no significant differences between patients with different clinical responses (**Figure 4A**). Dividing the patients into a response group (PR patients, *n* = 2) and a non-response group (SD/PD patients, *n* = 5), we quantitated the scores of 10 immune cell types from the pretreatment RNA-seq data (**Figure 4B**). The heat map also showed that monocytes were significantly elevated in the response group, while the others showed no clear pattern (**Figure 4B–D**). The immune microenvironment of PD patients also showed a deficiency of immune cells, especially T cells (**Figure 4B–C**). The differences in cell abundance between the reactive and non-reactive groups were compared, and monocytes were found to be significantly higher in the reactive group (**Figure 4E**). Monocytes are known to have phagocytic, antigen-presenting, and cytokine producing functions^37^, so the higher number monocytes might indicate better immunotherapy responses. However, this positive result needs to be validated using more samples.

Besides immunocytes, tumor tissues also have a certain percentage of infiltrating stroma cells, which have been reported to affect the immune microenvironment. It had been reported that the “activated” stroma subtype may describe the activated inflammatory stromal microenvironment, which has a shorter overall survival than normal stroma subtypes^38,39^. The 7 patients were clustered on the heat map according to the expression levels of stromal signature genes (**Figure 4F**). It was found that PR patients had decreased stromal gene expressions, while PD patients had active stromal gene expressions, thus PR patients had a normal stromal subtype while PD patients had an active stromal subtype. PR patients may therefore exhibit normal stromal subtypes while PD patients exhibit active stromal subtypes, suggesting that in sarcoma patients, an activated stromal subtype may indicate a poor response to immunotherapy.

#### The gene expressions of important tumor and immune associated signaling pathways

**Figure 5:**
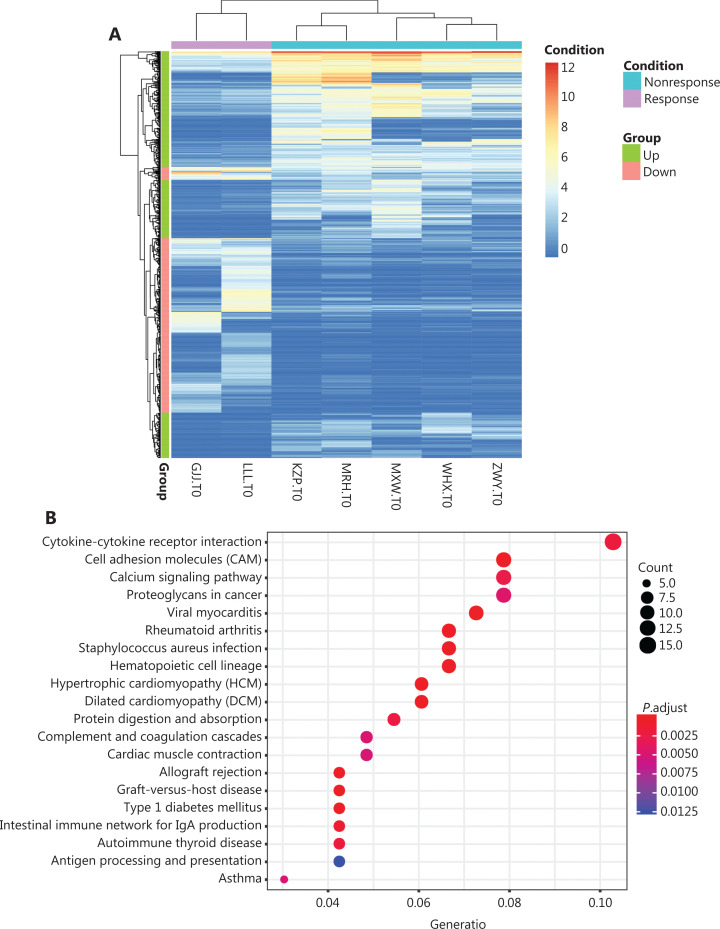

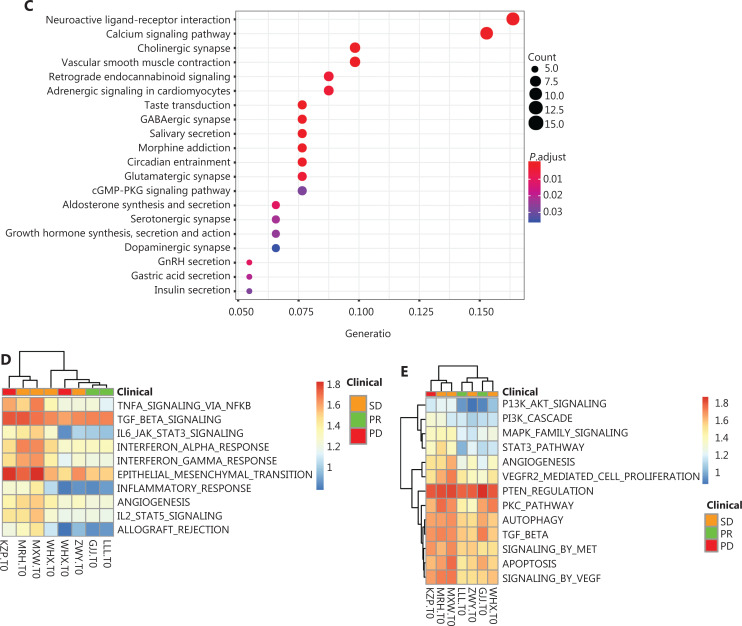
The analysis of transcriptome data. (A) The gene set enrichment analysis of the signal pathway. (B) The increasing expressions of genes mainly belong to cytokine-cytokine receptor interaction, cell adhesion molecules, the calcium signal pathway, and proteoglycans in cancer. (C) The decreased expressions of genes mainly belonged to neuroactive ligand-receptor interaction and the calcium signal pathway. (D) The gene set enrichment analysis of signal pathway-related immunity. The increased TGF-β signaling pathways had reverse correlations with anti-PD1 efficacies, while the decreased inflammation responses of signaling pathways had positive correlations with anti-PD-1 efficacies. (E) Key tumor-associated signaling pathways such as PI3K-AKT, PTEN, and MAPK showed no outstanding difference among different response patients.

Seven patients were analyzed for differences in gene expressions between efficacy groups at the RNA level before treatment (selected *P* < 0.05, |Fold change| >2) (**Figure 1**). There were 372 genes with increased prominent expressions and 318 with decreased prominent expressions (**Figure 5A**). We also performed enrichment analysis of differentially-expressed genes to identify the affected pathways. The genes with increased expressions mainly belonged to cytokine-cytokine receptor interactions, cell adhesion molecules (CAMs), calcium signaling pathways, and proteoglycans in cancer (**Figure 5B**), while genes with decreased expressions mainly belonged to neuroactive ligand-receptor interactions and calcium signaling pathways (**Figure 5C**). We then investigated the relationship between immune-related pathways such as TGF-β, inflammatory responses, TNFA signaling *via* NFKB, IL6-JAK-STAT3, and the epithelial-mesenchymal transition with the effect of anti-PD-1 therapy (**Figure 5D**). We found that an increase in the TGF-β signaling pathway was inversely correlated with anti-PD1 efficacy (**Figure 5D**), while a decrease in the inflammatory response signaling pathway was positively correlated with anti-PD1 efficacy (**Figure 5D**). However, most of the key tumor-related signaling pathways such as PI3K-AKT, PTEN, and MAPK did not show differences among patients with different responses (**Figure 5E**).

Some patients were treated with anti-PD-1 plus apatinib, and some studies reported that the antitumor effects of apatinib might be associated with VEGF, VEGFR2, angiogenesis, apoptosis, autophagy, EMT/MET, and multiple drug resistance (MDR) signaling pathways^40,41^; therefore, we also measured levels of these keys signaling pathways before treatment (**Table 6; Supplementary Figure S2A–M**). However, we did not find a significant pattern of these pathways. In the signal pathways of VEGF, AXL and ITPR3 showed low expressions in the response groups, while PAK3 and PRKCA showed high expressions in the response groups (**Supplementary Figure S2A**). In the signal pathway of VEGFR2, ITPR3 showed low expression in the response groups, and PRKCA showed high expression in the response groups (**Supplementary Figure S2B**). We also found that PRKCA showed increasing expressions in the VEGF, VEGFR2, and BioCarta-PKC groups, which belonged to the MDR signaling pathways (**Supplementary Figure S2A, S2B, S2H**). PRKCA is 1 of the PKC family members. This kinase has been reported to play roles in many different cellular processes, such as cell adhesion, cell transformation, cell cycle checkpoints, and cell volume control.

**Table 6:** Genes expression in the key signaling pathway

Class	Pathway name	Gene	UP/DOWN
VEGF	REACTOME_SIGNALING_BY_VEGF	AXL, ITPR3	UP
		PAK3, PRKCA	DOWN
VEGF2	REACTOME_VEGFR2_MEDIATED_CELL_PROLIFERATION	ITPR3	UP
		PRKCA	DOWN
ANGIOGENESIS	HALLMARK_ANGIOGENESIS	S100A4, LUM, VCAN	UP
APOPTOSIS	REACTOME_APOPTOSIS	TNFSF10	UP
		BCL2	DOWN
AUTOPHAGY	REACTOME_AUTOPHAGY	TUBA4A	UP
MET	REACTOME_SIGNALING_BY_MET	COL1A1, COL5A3, COL1A2, COL5A1	UP
EMT	TGF-beta_receptor_signaling_in_EMT	CGN	DOWN
Drug resistance related	BIOCARTA_PKC_PATHWAY	PRKCA	DOWN
	REACTOME_MAPK_FAMILY_SIGNALING_CASCADES	APBB1IP, FGF16, CAMK2A	UP
		NEFL, NRG3, PAK3, RASGRF1, NRTN	DOWN
	REACTOME_PI3K_AKT_SIGNALING_IN_CANCER	FFGF16, CD86, RAC2, CD28D	UP
		NRG3	DDOWN
	REACTOME_PI3K_CASCADE		
	REACTOME_PTEN_REGULATION		
	ST_STAT3_PATHWAY		

## Discussion

With the effectiveness of anti-PD-1 therapy in malignant melanomas and lung cancers, more attention has been directed to immunotherapy. Sarcomas are not included in the approved indications of anti-PD-1 agents, but its immunotherapy still needs further study. Our results showed that ORR_−12w_ was 22.2%, the DCR_−12w_ was 72.2%, the PFR_−12w_ was 49.8%, and the median PFS was 7.59 months. These results are consistent with previous reports on the efficacy of PD-1 inhibitors in sarcomas^15,18,42^. Our data suggested PD-1 inhibitors combined with anti-angiogenesis agents were novel and effective treatments for metastatic sarcomas after failure of conventional therapies. More importantly, we found that the ITH, monocyte ratio, stroma subtype, and the status of immune-associated signaling pathways may have correlations with anti-PD-1-based therapies.

Although PD-L1 expression (>50%) can serve as an indicator of pembrolizumab in NSCLC^43^, PD-L1 expression level alone cannot be used as an independent criterion for predicting the effectiveness of anti-PD-1 therapies in all tumors. In sarcomas, studies have evaluated the expression levels of PD-L1 in osteosarcomas and soft tissue sarcomas, but patients with high PD-L1 expressions did not achieve better outcomes after receiving immunotherapy^44–46^. The degree of PD-1 positivity in tumor-infiltrating lymphocytes, and PD-L1 expressions in tumor specimens from 105 cases of soft tissue sarcomas correlated with a poor prognosis and aggressive disease^47^. Furthermore, PD-L1 expression of the same sarcoma subtypes was not exactly the same in different trials^48,49^. This may be due to the standardization process of testing, different antibodies used for PD-L1 detection, the number of proprietary companion diagnostics, different samples used for specific sarcoma subtypes, and the lack of clear definitions of a “positive” tumor in terms of PD-L1 staining by immunohistochemistry (IHC)^50^. In prospective clinical trials, anti-PD-1 therapy for bone and soft tissue sarcomas resulted in minimal responses^14,15,51,52^. Furthermore, PD-L1 is a useful predictor of poor prognoses in patients with bone and soft tissue sarcomas, but is insufficient as a predictor of anti-PD-1 therapy efficacy^44,52^. Our results showed that only 3 out of 13 patients (23.07%) had positive PD-L1 expressions without sarcoma type tendencies. PD-L1 expression also had no significant correlation with the response to anti-PD-1 treatments in patients with sarcomas. As a result, more investigations will be necessary to determine whether PD-L1 expression can serve as a reliable biomarker for anti-PD-1 treatment in bone and soft tissue sarcomas.

Because the subtypes of sarcomas are numerous and complicated, it is important to determine which subtypes are sensitive to immunotherapy. Although extended cohort studies have shown that UPSs and ASPSs are sensitive subtypes to anti-PD-1 therapies^15^, our results suggested that leiomyosarcoma, osteosarcoma, and chondrosarcoma may also respond to anti-PD-1 therapies. Recent genomic studies have confirmed the different mutation burdens of different tissue types^47^. Many types of sarcomas in adults, as well as osteosarcomas in pediatric patients, have high mutation burdens but lack consistent targetable underlying events. The therapeutic effects of immunosuppressive checkpoint inhibitors on different subtypes of sarcomas differ^53–55^, with the differences between subtypes of sarcomas, including pathological alterations such as translocated genes, fusion genes, and mutation burden, suggesting that anti-PD-1 therapy is an option^53^. Studies have shown that most subtypes with a low mutation burden tended to be translocation-associated sarcomas, including Ewing sarcoma, rhabdomyosarcomas, and synovial sarcomas. However, the high mutation burden subtypes include osteosarcomas, leiomyosarcoma, and undifferentiated pleomorphic sarcomas^8^. TMB differences also include the genetic instability of some specific subtypes acquired during metastasis or recurrence^53^. The differences of subtypes in etiology and mutation burden result in differences of effectiveness.

The therapy based on or combined with PD-1 inhibitors, such as radiotherapy, chemotherapy, and anti-angiogenesis, needs additional study. In our data, a PD-L1 positive LMS patient received PR at the same time of radiotherapy. The efficacy was the result of monotherapy against PD-1, or the synergistic effect of radiotherapy. The “abscopal effect” of radiotherapy may increase the burden of mutation and stimulate immunity. The expression of PD-L1 in human sequence-tagged sites (STS) and tumor-associated macrophages (TAMs) can increase after preoperative radiotherapy^53^. Radiation therapy can induce increased antigenic expression, release pro-inflammatory cytokines that recruit immune cells, promote antigen cross-presentation, and induce tumor expression of death receptors^54,55^. Considering the synergistic effects of anti-PD-1 treatment and radiotherapy, the reasons mentioned above should not be excluded. We should therefore consider PD-1 inhibitor-based therapy, especially anti-PD-1 therapy combined with radiotherapy, as a promising and effective strategy. This combination may produce synergistic effects. At the same time, imRecist should be used to evaluate the effect of immunotherapy. Treatments of PD-L1 inhibitors combined with angiogenesis inhibitors have resulted in increased interest in this process.

In a 68-year-old female patient with osteosarcoma, the results of gene sequencing from biopsy tissue could not explain the treatment response at the molecular level. Gene sequencing results showed that the mutation load was 2.3 mutations per megabase (muts/mb), and IHC results showed that PD-L1 was negative. These results suggested that the patient may not have responded to anti-PD-1 therapy. However, the efficacy of anti-PD-1 treatment has been confirmed clinically. We also screened the role of PTEN InDel in the PI3K signal pathway by whole genome sequencing of the patient. The change of PTEN in the PI3K signal transduction pathway can change the tumor microenvironment. It has been reported that the relationship between PTEN deletion and immunotherapy resistance suggested that PTEN gene deletion may be one of the driving mechanisms of tumor resistance to PD-1/PD-L1 inhibition. Therefore, even if the expression of PD-L1 is negative, TMB is low, MSI is stable, and the PTEN InDel is positive in sarcomas, it is difficult to explain why this osteosarcoma patient obtained the PR effect.

We found that the expressions of TMB/TNB, MSI, HLA-LOH, and PD-L1 could not be used as independent predictors of anti-PD-1 therapy. Immunotherapy efficiency and bioinformatics analysis showed that the levels of ITH in PD patients was higher than that in PR and SD patients. Furthermore, PR patients may have a higher percentage of immune cells, especially monocytes. The expressions of active matrix gene decreased in PR patients and increased in PD patients. Based on the enrichment analysis of RNA sequencing data, it was found that TGF-β, an increase of the signal pathway was negatively correlated with the efficacy of anti-PD-1, while a decrease of the inflammatory signal pathway was positively correlated with the efficacy of anti-PD-1. The status of ITH, monocyte ratio, stromal subtypes, and immune related signaling pathways may therefore be correlated with the efficacy of anti-PD-1 treatments for sarcoma, but more samples and analyses are needed to confirm these results. Our ongoing clinical trial of NTC04126993 in the treatment of advanced bone and soft tissue sarcomas in China is therefore of great importance.

Although our study confirmed that PD-1 inhibitors were effective in the treatment of advanced sarcomas, this observational study had shortcomings and deficiencies. First, this was a retrospective analysis of anti-PD-1 treatments. There were not enough cases to be divided into groups according to the use of monotherapy or the combination of PD-1 inhibitors. However, these results provided us with some ideas, such as combination therapy strategies for sarcomas, but more prospective clinical trials are needed to confirm these findings. Second, due to uncontrollable factors, some patients did not review on time, so the evaluations of efficacy and adverse events were insufficient. In addition, due to economic problems, we were unable to obtain gene sequencing results for all patients, and we could not compare and predict treatment differences at the molecular level. Even in drug-resistant patients, we could not compare point mutations or gene sequence changes. In addition, only 8 patients had gene sequencing data, and the others had no pathological tissue because surgery was performed in other hospitals. The results of PD-L1 expression should only be considered when using different PD-L1 antibodies. In addition, the cut-off point for positive outcomes must be considered.

## Conclusions

In this retrospective study of the largest cohort of sarcomas in China, we reported that PD-1 inhibitor-based therapies for advanced sarcomas were effective, suggesting that PD-1 inhibitor combined with anti-angiogenic drugs is a new and effective treatment for metastatic sarcomas after the failure of chemotherapy. Most importantly, we showed that although PD-L1 expression, MSI, TMB/NB, and HLA-LOH were difficult to use as independent biomarkers for anti-PD-1 therapy in metastatic sarcomas, ITH, monocyte ratio, and interstitial subtypes, and the status of immune related signaling pathways may be related to the efficacy of anti-PD-1 therapy in sarcomas. Although there are many questions still to be answered, we look forward to the results of the NCT04126993 clinical trial for the treatment of advanced sarcoma, in order to identify more effective treatment options for patients with sarcoma.
