# Supplementary material for: The efficacies and biomarker investigations of anti-programmed death-1 (anti-PD-1)-based therapies for metastatic bone and soft tissue sarcoma
Source: Cancer Biol Med. 2021 Nov 25;19(6):910–30. doi: 10.20892/j.issn.2095-3941.2021.0270 (PMC9257312; doi:10.20892/j.issn.2095-3941.2021.0270)
Supplement: Supplementary file 1 [file cbm-19-910-s001.pdf]

Supplementary materials

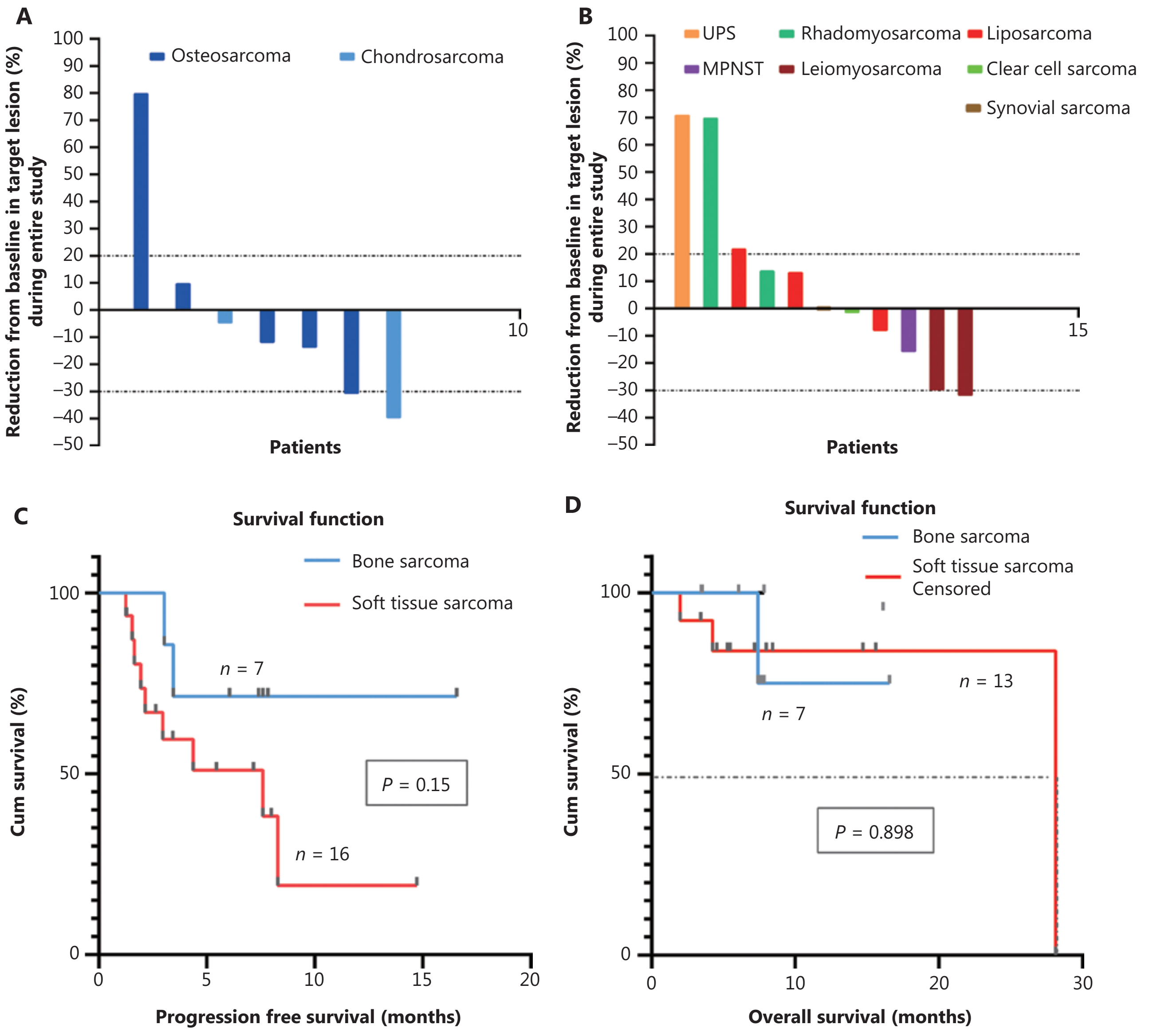

Figure S1 Maximum change in target lesion size and survival of bone sarcomas and soft tissue patients with stage IV sarcomas treated with anti-PD-1-based therapy. Maximum change in target lesion size in patients with (A) stage IV bone sarcomas and (B) stage IV soft tissue sarcomas. There was no significant difference between patients with bone sarcomas and those with soft tissue sarcomas in terms of (C) median progression-free survival or (D) median overall survival.

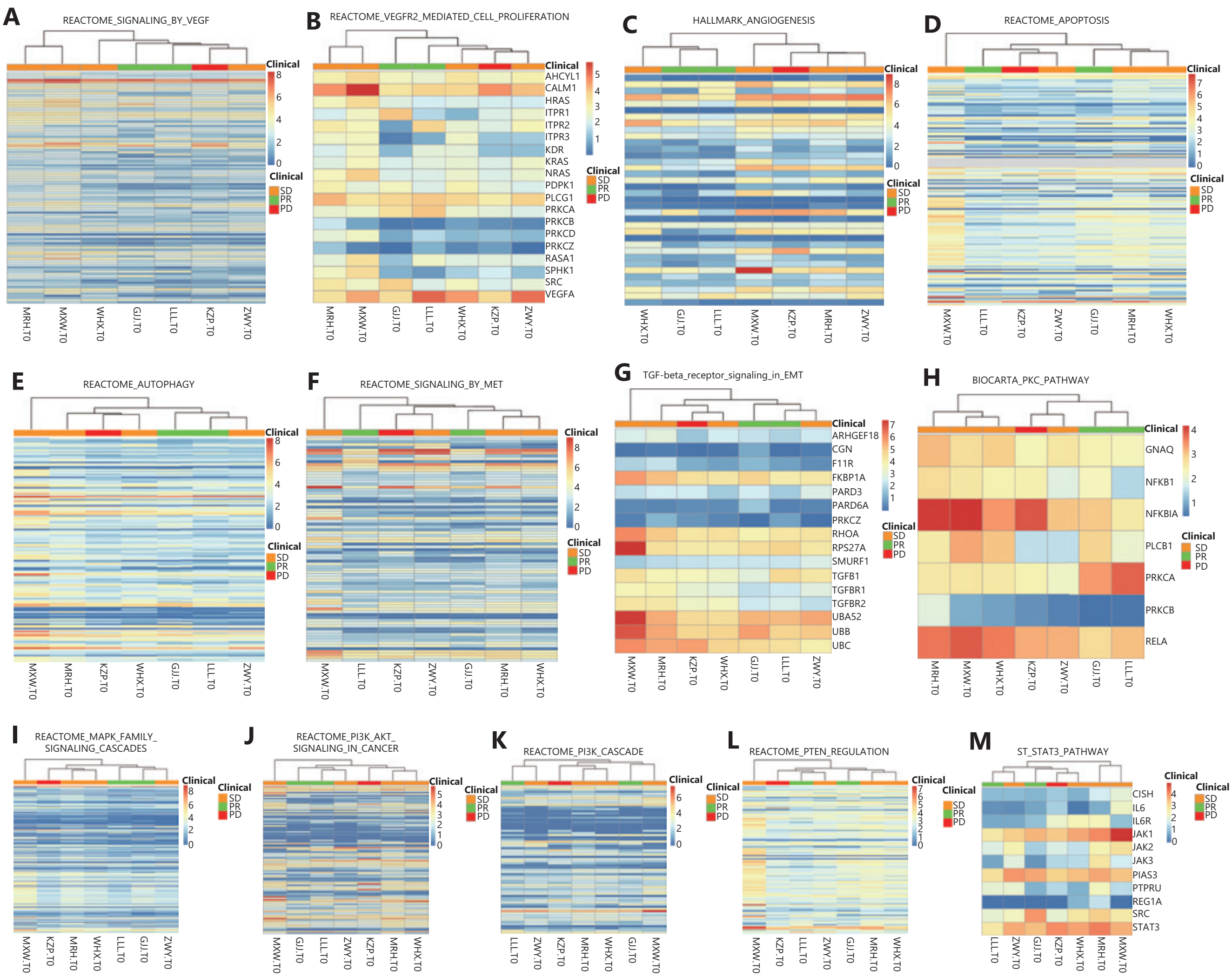

Figure S2 The gene expressions of other key signaling pathways. (A) The gene expression heat map of the VEGF signaling pathway. The *AXL* and *ITPR3* genes showed decreasing expressions, and those of *PAK3* and *PRKCA* showed increasing expressions in the response group (SD + PR). (B) The VEGFR2 signaling pathway. *ITPR3* showed decreasing expressions, and *PRKCA* showed increasing expressions in the response group. (C) The angiogenesis-related signaling pathway. *S100A4, LUM*, and *VCAN* showed decreasing expressions in the response group. (D) The apoptosis-related signaling pathway. *TNFSF10* showed decreasing expression in the response group, and *BCL2* showed increasing expression in the response group. (E) The autophagy-related signaling pathway. *TUBA4A* showed decreasing expression in the response group. (F) The MET signaling pathway. *COL1A1, COL5A3, COL1A2*, and *COL5A1* showed decreasing expressions in the response group. (G) The epithelial-mesenchymal transition signaling pathway. *CGN* showed increasing expression in the response group. (H–M) The gene expressions of multiple drug resistance-related signaling pathways, including PKC, the MAPK family, PI3K-AKT, PI3K, PTEN, and the ST-STAT3 signaling pathway.

A leiomyosarcoma patient treated with anti-PD-1 and radiotherapy

A 40-years-old female was diagnosed with mediastinal leiomyosarcoma in 2011. After right inferior lobe mass resection, the patient was treated concurrently with radiotherapy and chemotherapy (DDP+TAX liposome) (**Supplementary Figure S3A**). After treatment, metastatic sites appeared in the back of the patient, which were completely resected. During the follow up, PET-CT showed there were metastases at multiple sites, including the brain, lungs, liver, and adrenal gland. She then received anti-PD-1 therapy (pembrolizumab) combined with macro-fractionated radiotherapy. The sites of radiotherapy included the brain and lung. After the second cycle of anti-PD-1 treatment, metastases in the lung, liver, and brain were enlarged and the overall increase in the lesion size was nearly 20%, resulting in a determination of PD (**Supplementary Figure S3B, C, F, G**). However, after four cycles of treatment, all target lesions progressively shrank by nearly 30% (**Supplementary Figure S3D, E, H, I**), compared to the baseline. The previous progress could be considered as pseudo-progress. But unfortunately, 11 cycles later, a new metastatic lesion appeared in the left fourth rib, which led to the evaluation of the patient as PD, even though some of the metastatic tumors were still decreased or stable. After consultation with the sarcoma multiple team, the patient was recommended to continue receiving anti-PD-1 therapy and concurrent radiotherapy, but the disease still progressed. She then received best support care treatment with regular follow-ups, until death from infections at multiple sites (**Figure 3A**). Unfortunately, DNA sequencing was not performed on this patient, so we were unable to assess the reasons for the failure of anti-PD-1 therapy at the gene level.

Osteosarcoma patients treated with anti-PD-1 monotherapy

The 68-year-old female patient was diagnosed with osteosarcoma involving the right ilium and adjacent sacrum. She was treated with chemotherapy (ADM+DDP+MTX+IFO). When the chemotherapy failed, the patient received mono-PD-1 inhibitor (pembrolizumab) therapy. After 4 courses of anti-PD-1 treatment, CT images showed that destructions of the right ilium and adjacent sacrum were localized and resulted in osteosclerosis. Meanwhile, the soft tissue mass became smaller and calcified (**Supplementary Figure S4A, C**). PET-CT images also showed a significant decrease of the SUV, from 13.9 before treatment (**Supplementary Figure S4B**) to 2.6 (**Supplementary Figure S4D**). The symptom of self-reported pain was relieved, and the activity was significantly improved. The maximum diameter of the metastatic site in the left lung also shrank from 1.5 cm to 1.1 cm (**Supplementary Figure S4E–F**).

DNA sequencing of the biopsy tissue before chemotherapy indicated that the tumor mutation burden (TMB) was 2.3 Mutations/megabase and the microsatellite was stable. The immunohistochemistry results of PD-L1 expression were negative (**Supplementary Figure S4H**). The patient's response to pembrolizumab was unexpected because the above reference tests appeared to not support immunotherapy. Using whole exon sequencing, we also screened the PTEN indel in PI3K signaling. As a well-known driver gene in tumors, the abnormality of the mTOR signaling pathway in this patient indicated that when the patient's disease progressed, mTOR inhibitors may be candidate treatment options (**Supplementary Figure S4I**). In addition, it has been reported that the loss of PTEN may modify the tumor microenvironment[1,2].

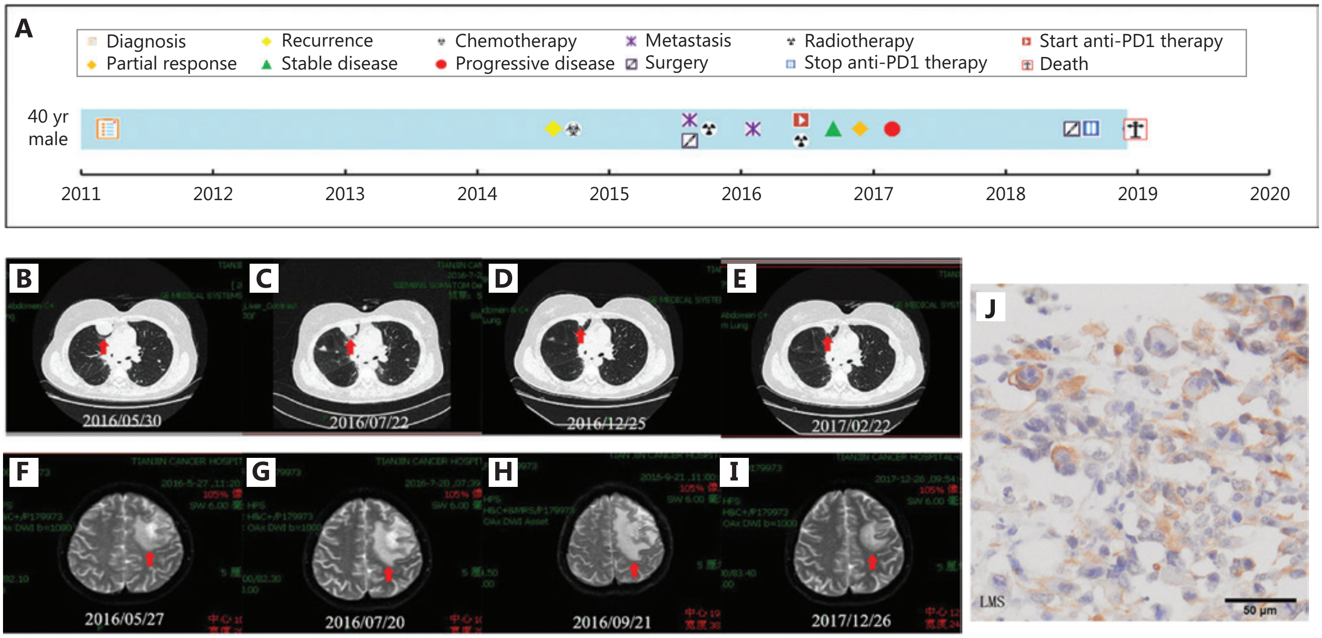

Figure S3 Treatment of the leiomyosarcoma patient and the target lesion response to pembrolizumab. (A) Details of the clinical treatment course. Before the patient received anti-PD-1 (pembrolizumab) therapy combined with macro-fractionated radiotherapy, the patient received chemotherapy (DDP+TAX liposomes, ADM) and radiotherapy. The overall survival was 28.12 months. Because of a new metastatic site in the rib, the patient was assessed as PD. (B–E) The response of the target lesions to pembrolizumab in the lung. Computed tomography revealed decreased tumor sizes after pembrolizumab treatment. (B) Before pembrolizumab treatment (May 30, 2016), the lung metastatic site was 2.5 × 3.2 cm. (C) After pembrolizumab treatment (June 22, 2016), the lung metastatic site was 2.7 × 3.7 cm. (D) After pembrolizumab treatment (November 25, 2016), the lung metastatic site was 1.7 × 1.5 cm. (E) After pembrolizumab treatment (February 22, 2017), the lung metastatic site was 2.0 × 1.0 cm. (F–I) The response of target lesions in the brain to pembrolizumab. Computed tomography revealed decreased or increased tumor size after pembrolizumab treatment. (F) Before pembrolizumab treatment (May 27, 2016), the brain metastatic site was 2.52 × 0.1 cm. (G) Before pembrolizumab treatment (July 20, 2016), the brain metastatic site was 2.6 × 3.2 cm. (H) Before pembrolizumab treatment (September 21, 2017), the brain metastatic site was 2.4 × 2.0 cm. (I) Before pembrolizumab treatment (December 27, 2017), the brain metastatic site was 1.7 × 1.7 cm. Compared with the baseline state and after treatment, the previous progress of the disease was considered as pseudo-progress. (J) The PD-L1 expression was positive using immunohistochemical staining.

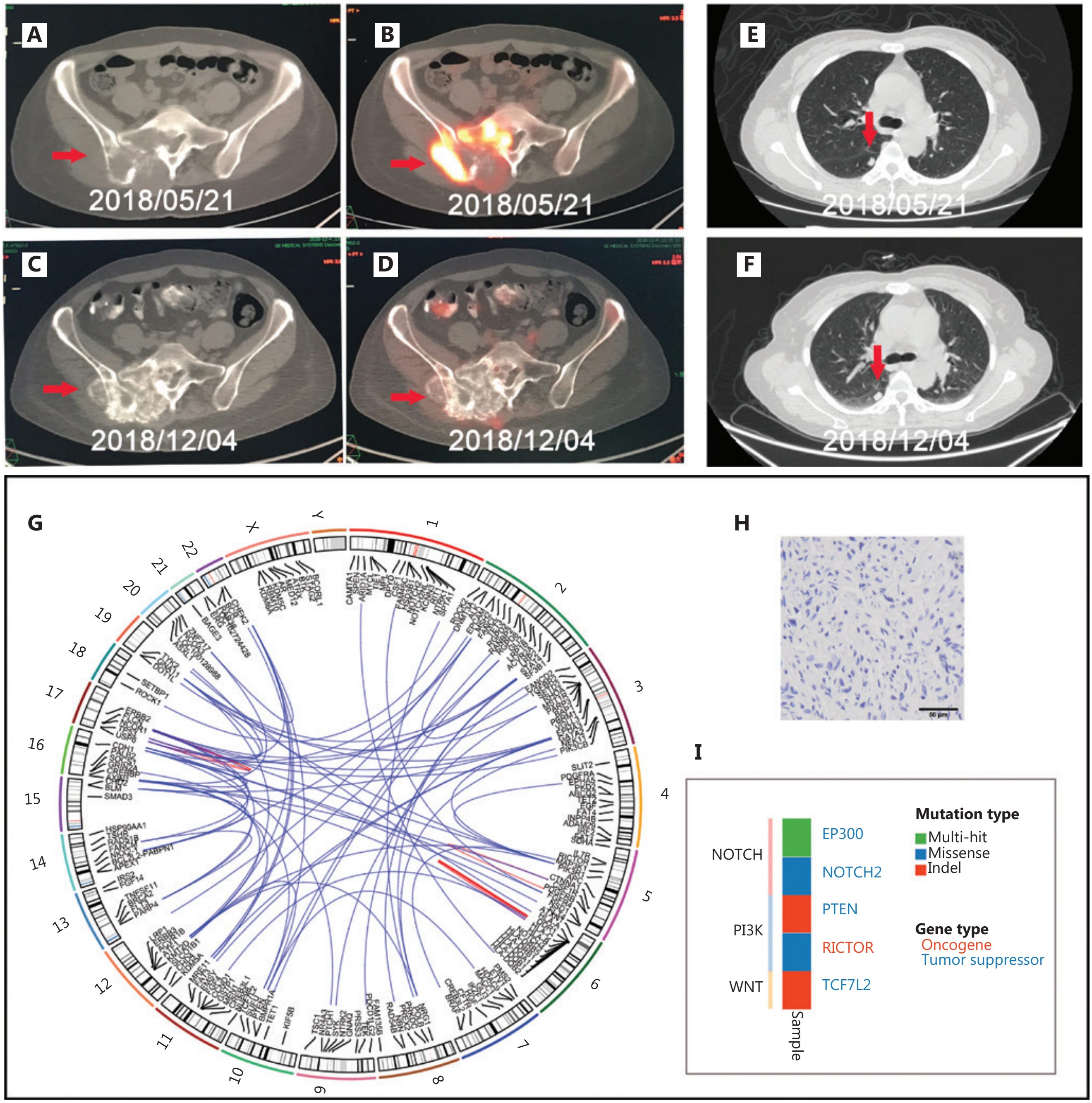

Figure S4 The response of the osteosarcoma patient to PD-1 inhibitor treatment and whole exon sequencing before anti-PD-1 therapy. (A, C) The response of target lesions in the right ilium and adjacent sacrum to treatment. Positron emission tomography (PET) revealed a changed primary tumor lesion after pembrolizumab treatment. (B, D) PET-computed tomography showed abnormal radioactivity concentration with a SUV value change from 13.9 to 2.6 and no other metastatic foci. (E, F) The response of target lesions in the lung to anti-PD-1 treatment. (E) Before treatment (May 21, 2018), the maximum diameter was 1.5 cm. (F) After treatment (December 04, 2018), the maximum diameter shrank to 1.1 cm. (G) The relationships among somatic mutations, on the basis of connected pairs, which were verified by experiments (credibility > 0.7) in the String database. (H) The intratumoral heterogeneity test result of PD-L1 expression was negative. (I) The selected somatic mutations were in different signaling pathways.

Supplementary references

1. Cretella D, Digiacomo G, Giovannetti E, Cavazzoni A. PTEN Alterations as a potential mechanism for tumor cell escape from PD-1/PD-L1 inhibition. Cancers. 2019; 11: 1318.
2. Stefano S, Giovanni S. The PTEN tumor suppressor gene in soft tissue sarcoma. Cancers. 2019; 11: 1169.

Criteria

Inclusion Criteria:

- Patients voluntarily joined the study, signed informed consents, and had good compliance.
- Pathologically confirmed patients with unresectable sarcomas (except GIST), clinical stage using the American Cancer Research Joint Committee (AJCC) TNM staging criteria. At least 1 double-path measurable lesion according to CT or MRI.
- At least one chemotherapy regimen (containing an anthracycline) was used to treat patients with disease progression or intolerance according to the solid tumor efficacy evaluation criteria (RECIST 1.1).
- Clear cell sarcoma and alveolar soft tissue sarcoma can be directly observed in the group without chemotherapy.
- An age of 14~75-years-old, PS score of 0~2, with an expected survival period > 3 months.
- All acute toxic reactions caused by previous anti-tumor treatment or surgery were relieved to 0–1 before screening (according to NCI CTCAE, Version 4.03) or to the level specified by the enrollment/exclusion criteria (alopecia, etc., except for toxicity that did not pose a safety risk to the patient).
- There were sufficient organ and bone marrow functions, defined as follows:
  - Blood routine (no blood transfusion within 14 days before treatment, no use of G-CSF, no use of drugs for corrections), neutrophil count ≥ 1,500/mm^3 (1.5 × 109/L); platelet count ≥ 100,000/mm^3 (100 × 109/L); hemoglobin ≥ 9 g/dL (90 g/L).
  - Blood chemistry, serum creatinine ≤ 1.5× of the upper limit of normal (ULN) or creatinine clearance (Cockroft-Gault formula) ≥ 60 mL/min; total bilirubin ≤ 1.5 × ULN; aspartate aminotransferase or alanine aminotransferase levels ≤ 2.5 × ULN, liver metastases should be ≤ 5× ULN.
  - Coagulation, international normalized ratio ≤ 1.5, prothrombin time and activated partial thromboplastin time ≤ 1.5 × ULN.
  - Urine routine, urine protein < 2+; if the urine protein was ≥ 2+, the 24 h urine protein quantitation showed that the protein must be ≤ 1 g.
  - Thyroid function, Thyroid stimulating hormone ≤ ULN; if abnormalities should consider T3 and T4 levels. T3 and T4 levels could be selected.
- Female subjects of childbearing age must undergo a serum pregnancy test within 7 days prior to treatment with negative results, and were willing to use a medically recognized effective contraceptive measure during the study period and within 3 months after the last administration of the study drug (e.g., intrauterine devices, contraceptives, or condoms); for male subjects whose partners were women of childbearing age, surgical sterilization was required, or an effective method of contraception was recommended during the study period and within 3 months after the last study administration.
- With my consent and signed informed consent, I am willing and able to follow the planned visits, research treatments, laboratory tests, and other testing procedures.

Exclusion Criteria:

- The following treatments were received within 4 weeks of treatment.
  - Radiotherapy, surgery, chemotherapy, immunization, or molecular targeted therapy for tumors; other clinical research drugs; and vaccination using live attenuated vaccines.
- Previously received treatment with PD-1/PD-L1/CTLA-4 antibody or VEGFR single target/multi-target inhibitor.
- Surgery and/or radiation therapy for soft tissue sarcomas was planned during the study (regardless of < 5% of the bone marrow area).
- Imaging diagnosis of central nervous system tumors.
- Immune-suppressing drugs were used within 14 days prior to initiation of treatment, excluding nasal and inhaled corticosteroids or physiological doses of systemic steroid hormones (no more than 10 mg/day of prednisolone or an equivalent physiological dose of other corticosteroids).
- There was any active autoimmune disease or a history of autoimmune disease, including but not limited to autoimmune hepatitis, interstitial pneumonia, uveitis, enteritis, hepatitis, pituitary inflammation, vasculitis, nephritis, hyperthyroidism, or hypothyroidism; subjects with vitiligo or asthma that had been completely relieved in childhood and currently did not require medical intervention were

included, or a history of allogeneic organ transplantation or a history of allogeneic hematopoietic stem cell transplantation.

- Severe infections (such as intravenous infusion of antibiotics, antifungal, or antiviral drugs) within 4 weeks prior to treatment, or unexplained fever > 38.5 °C during screening/first administration.
- High blood pressure, and excellent control without antihypertensive medication (systolic blood pressure > 140 mmHg or diastolic blood pressure > 90 mmHg).
- There were significant clinically significant bleeding symptoms or clear bleeding tendency within 3 months before treatment, such as gastrointestinal bleeding, hemorrhagic gastric ulcer, baseline fecal occult blood ++ and above, vasculitis, etc. Or venous/venous thrombosis events occurred within 6 months prior to treatment, such as cerebrovascular accidents (including transient ischemic attacks, cerebral hemorrhage, cerebral infarction), deep vein thrombosis, and pulmonary embolism; or disorders that required long-term anticoagulant therapy with warfarin or heparin, or long-term antiplatelet therapy (aspirin ≥ 300 mg/day or clopidogrel ≥ 75 mg/day).
- There was active heart disease 6 months before treatment, including myocardial infarction, severe/unstable angina. Echocardiography left ventricular ejection fraction < 50%, poorly controlled arrhythmia (including QTcF interval men > 450 ms, women > 470 ms).
- Any other malignant tumor was diagnosed within 3 years prior to treatment, except for adequately treated basal cells or squamous cell skin cancer or cervical carcinoma *in situ*.
- The patient was known to be allergic to the study drug or any of its excipients, or to have a severe allergic reaction to other monoclonal antibodies.
- Human immunodeficiency virus infection, active hepatitis B (HBV-positive and HBV DNA ≥ 500 IU/mL), hepatitis C (positive hepatitis C antibody and a higher detection limit of HCV-RNA than analytical methods).
- At the discretion of the investigator, there were concomitant diseases (such as poorly controlled hypertension, severe diabetes, neurological, or psychiatric disorders, etc.) that seriously compromised the safety of the subject, and which may have confused the findings, or affected the patient's completion of the study. Any other similar situation.
